# Factors Associated With Uptake of Breast Cancer Screening Among Catholic Nuns in Lake Zone, Tanzania

**DOI:** 10.1155/2024/5024392

**Published:** 2024-10-12

**Authors:** Gotfrida Marandu, Rose Laisser, Kija Malale, Peter Rambau

**Affiliations:** ^1^Archbishop Anthony Mayala School of Nursing, Catholic University of Health and Allied Sciences Bugando, Mwanza, Tanzania; ^2^Department of Pathology, Catholic University of Health and Allied Sciences Bugando, Mwanza, Tanzania

**Keywords:** acceptability, breast cancer screening, Catholic nuns, uptake

## Abstract

**Background:** Breast cancer is the most common cancer among women worldwide. Breast cancer screening programs are widely promoted because of their effectiveness in the early detection of cancer. However, a significant proportion of eligible Catholic nuns in the Lake Zone of Tanzania remain underscreened. This study is aimed at investigating the factors associated with breast cancer screening uptake among Catholic nuns in Lake Zone, Tanzania.

**Methods:** This was a cross-sectional study design among 385 catholic nuns. Simple random sampling was deployed to enrolled catholic nuns, the ODK collect v2023.2.4 was electronically used to collect data. Binary logistic regression was used to assess the factors associated with the uptake of breast cancer screening. Statistical analysis was performed using STATA 18.0, with a significance level set at a *p* value less than 0.05.

**Result:** The prevalence uptake of breast cancer screening (i.e., breast self-examination, clinical examination, or mammography examination) was 64% (*n* = 247, 95% CI, 59.3%–68.9%). A total of 57.4% had inadequate knowledge on the uptake of breast cancer screening (*n* = 221, 95% CI, 52.4%–62.4%). Also, the study found that 61.3% (95% CI, 56.4%–66.2%) of Catholic Nuns have negative attitudes towards the uptake of self-breast examination among Catholic nuns. The findings revealed that 55.6% (*n* = 133, 95% CI, 50.6%–60.6%) and 52.7% (*n* = 52.7%, 95% CI, 47.7%–57.7%) of Catholic nuns negatively accept breast cancer screening and self-breast examination, respectively. Nuns aged above 60 years were less likely to not perform BCS with a COR of 0.62 (95% CI, 0.39–0.97). Also, Catholic nuns who are in the nonhealth field are more likely to not perform BCS with a COR of 1.71 (95%, 1.07–2.74). Likewise, Catholic nuns who had negative acceptability of the Self-breast examination were more likely to not perform BCS with an AOR of 1.65 (95% CI, 1.07–2.55).

**Conclusion:** A study found a low uptake of breast cancer screening among Catholic nuns. This highlights the need for breast health intervention programs within religious congregations to address misconceptions and promote early detection.

**Summary**

• A recent study conducted in Tanzania's Lake Zone investigated breast cancer screening in Catholic nuns. Breast cancer screening is important for early detection of breast cancer. The study found more than half (64%) of the 385 nuns surveyed reported undergoing breast cancer screening.
• This low rate may be attributed to a lack of knowledge. More than half (57%) of the nuns reported insufficient information about breast cancer screening. Additionally, negative attitudes towards screening were prevalent, with over 61% of the nuns expressing discomfort during the process.
• This study suggests that religious communities play a vital role in improving screening rates. However, breast health intervention programs within these communities could address misconceptions and encourage nuns to prioritize breast cancer screening. Early detection is crucial for successful treatment, and these programs can empower nuns to manage breast health.

## 1. Introduction

Breast cancer (BC) is the most common cancer and the primary cause of cancer-related deaths among women globally [1]. In 2020, an estimated 2.3 million new cases were diagnosed (representing 1 in 4 new cancer cases), with 685,000 deaths (1 in 6 deaths). According to WHO, incidence rates vary significantly across regions [2]. Despite advancements in diagnosis and treatment, BC remains a major threat to women's health, particularly in low- and middle-income countries (LMICs) [3]. These regions disproportionately suffer, accounting for over 60% of new cases diagnosed in 2020 [4]. The burden of BC extends beyond individual suffering, creating substantial morbidity and economic strain on healthcare systems, societies, and economies [2, 4].

Sub-Saharan Africa (SSA) faces a stark disparity in BC survival rates compared to developed nations. While developed countries like the United States boast an 86% 5-year survival rate, SSA struggles with a rate below 40% [5]. This concerning gap is projected to widen, with an estimated 19.3 million women, primarily from SSA, likely to be diagnosed with BC by 2025 [6]. Several factors contribute to this disparity, including limited access to early detection programs, multimodality treatments, and adequate healthcare facilities [7]. This signifies that the rate of prevalence of BC among nuns globally is high [8]. A study done by Britt, K. et al. in Verona, Italy estimated that cancer was 5 times more frequent among Sisters (nuns) than other women and ascribed this excess to an increased risk of BC [8].

In Tanzania, BC is the second most common cancer, accounting for 14.4% of new cases, and the second leading cause of cancer-related deaths among women [3, 9]. Alarmingly, BC incidence in Tanzania is projected to rise up to 82% by 2030 [10].

The early detection of BC plays a key role in successful treatment and in improving patient outcomes. However, various factors, particularly in resource-limited settings, can hinder the early detection of BC, including low uptake of BC screening (BCS) programs [11].

The early detection of BC is crucial for successful treatment. However, various challenges impede this [12]. Scarce diagnostic equipment, such as mammography machines and ultrasound technology, significantly restrict the ability to diagnose BC early stage [13]. Furthermore, the lack of organized screening programs has hindered awareness and accessibility. Despite these limitations, BCS programs featuring self- and clinical-breast examinations as well as mammograms (where available) have been advocated as a critical step towards creating awareness and promoting timely diagnosis and treatment [14]. Without proactive systems in place to promote and facilitate regular screenings, women may not be aware of the importance of early detection and may face difficulties in accessing these services [15]. Studies showed that when combined with appropriate treatment, early detection through BCS activities that include breast self-examination (BSE), clinical breast examination (CBE), and mammography has been shown to decrease cancer mortality rates due to BC by 25%–30% [16, 17]. As described by the Centers for Disease Control and Prevention (CDC) [18], BCS entails checking a woman's breasts for any symptoms or signs of BC [19]. Every individual should be informed of the BCS approach available to them. The common methods for detecting BC in its early stage are BSE, CBE, and mammography [20]. Mammography screening is expensive, requiring a significant amount of financial and human resources, and is thus not feasible in developing nations [21]. The BSE is a means for a woman to examine her breast for changes such as discharge, lumps, or thickenings, as well as the early diagnosis of breast lumps. It is a free technique, safe, requires no equipment, and can be done in privacy [22] This information is essential for making informed decisions regarding the screening process and whether it is suitable for the patient. This reflects the shared decision-making process and person-centre care model. Although BCS does not cure or prevent BC, it is a valuable tool for its early detection. Early detection allows more effective treatment options and potentially improved outcomes [17].

Catholicism in Tanzania faces disparities in the incidence of BC. Studies have suggested a higher prevalence of BC in nuns than in the general population. However, BCS uptake among nuns remains low [23, 24]. This highlights a critical knowledge gap regarding how religious beliefs and cultural norms within these communities influence screening practices how religious beliefs and how they influence screening practices [25]. Despite ongoing health campaigns, many nuns are still being diagnosed with BC at advanced stages [26]. This study explores this gap by examining the factors associated with BCS uptake among Catholic nuns in Lake Zone, Tanzania. By establishing these factors, we aimed to develop targeted interventions to improve the screening rates in this vulnerable population.

## 2. Materials and Methods

### 2.1. Study Design

This cross-sectional study was conducted among Catholic nuns aged 20 years and above in the Lake Zone of Tanzania from June to November 2023. We focused on the four highly populated congregation centers of Catholic nuns within the lake zone. Inclusion criteria were Catholic nuns aged 20 and above who were living in convents at the time the data were collected with no previous cancer diagnosis and who were ready to participate in the study.

### 2.2. Sample Size and Sampling Procedure

The study sample size was calculated using Cochran's formula for estimating a single proportion [27] with a desired level of precision of 5% significance level and a prevalence of BC of 34.4% [28]. This sample size was adjusted to account for an anticipated 10% nonresponse rate. Further, the study assumes an infinity population. The calculation is shown below.

 $$\begin{array}{c} n=\frac{Z^{2}p\left(1-p\right)}{e^{2}} \end{array}$$

where *Z* = *Z*-score at 95% confidence level (i.e., *Z* = 1.96). *e* is the precision level. *p* is the prevalence of BC (i.e., *p* = 34.4%).

 $$\begin{array}{c} n=\frac{{\left(1.96\right)}^{2}0.344\left(1-0.344\right)}{{\left(0.05\right)}^{2}} \end{array}$$

 $$\begin{array}{c} n=346.764 \end{array}$$

Adjusted by 10% nonresponse rate,

 $$\begin{array}{c} n=347÷0.90≅385 \end{array}$$

Therefore, the study randomly recruited 385 Catholic nuns residing in the Lake Zone of Tanzania to participate in the study.

In addition, a simple random sampling technique was used to select study participants. We identified all eligible nuns over 20 years of age with no prior cancer and assigned them unique identification numbers (IDs). These IDs, along with a few blanks for potential ineligibility, were written on paper slips and thoroughly mixed in a container. Our goal was to recruit a minimum of 385 participants. We drew IDs one at a time, without replacement, from each convent until we reached our target number for that convent (or encountered a blank slip). This approach ensured the random and representative selection of nuns from the entire Lake Zone.

## 3. Data Collection Instrument and Procedure

A structured questionnaire assessed the participants' sociodemographic characteristics, knowledge, attitudes, and practices related to BCS methods, acceptability, and uptake. The questionnaire was developed based on relevant literature [29–34] and was tailored to this study's objectives to assess factors associated with the uptake of BCS among Catholic nuns. The questions were divided into four sections, intended to capture the demographic information of respondents: Section 2 assessed respondents' knowledge of BC, Section 3 assessed respondents' attitudes towards BCS, and Section 4 assessed respondents' acceptance of BCS. Thereafter, the questionnaire was translated from English to the Kiswahili language which most of the nuns speak. All the data were self-reported and collected. Furthermore, an open data kit (ODK) Collect v2023.2.4 was used to capture data electronically. Through this, it helps to ensure the data quality. Likewise, the tool was pretested and checked for internal validity.

### 3.1. Pretest

To ensure that the questionnaire accurately captured the intended data, we employed a careful, two-step process. First, we established content validity by having a panel of four experts review the questionnaire for comprehensive coverage of the target concepts and clarity of language suitable for the target audience. Second, we conducted a pretest with 35 participants to identify any potential issues with the questionnaire's wording, instructions, or missing elements. This combined approach of content validity assessment and pretesting strengthened the overall validity of the questionnaire.

### 3.2. Internal Consistency

Cronbach's alpha was used to assess the internal consistency of the questionnaire. Cronbach's alpha coefficient was used to assess the internal consistency of the questions used to assess attitudes towards BCS (9 items), acceptability of BCS (10 items), and acceptability of BC examination (6 items). Cronbach's alpha yielded satisfactory results for attitudes towards BCS (0.7120), acceptability of BCS (0.7748), and acceptability of BC examination (0.7389). All reliability coefficients were above the threshold of 0.70, implying that the data were reliable for determining factors associated with BCS [35, 36].

### 3.3. Variable Measurements

#### 3.3.1. Uptake of BCS

The uptake of BCS was assessed by the question “If ever performed BCS, i.e., BSE, CBE, and mammography examination” and was binary measured (i.e., 0 = *yes* and 1 = *no*).

#### 3.3.2. Knowledge of BCS

The respondents' knowledge of BCS was assessed using twelve 12-item nominal scales. We calculated the overall score as the sum of the 12 items within a range of 1–12. Then, the mean (4.28) was calculated as well used as the cutoff to establish the dichotomous variable that respondents with a score above 4.28 were regarded to have adequate knowledge (labelled “0”), while below and equal to 4.28 was regarded to have inadequate knowledge (labelled “1”).

#### 3.3.3. Attitude Towards BCS

The attitude was assessed using a 9-item 5-Likert scale measurement. We calculated the overall score as the sum of the 9 items within the range of 1–45. Then, the mean (17.09) was calculated as well as used as the cutoff to establish a binary variable that respondents with a score above 17.09 were regarded to have positive attitudes (labelled “0”), while those below and equal to 17.09 were regarded to have a positive attitude (labelled “1”).

#### 3.3.4. Acceptability of BCS

The acceptability of BCS was assessed using a 10-item 5-Likert scale measurement. We calculated the overall score as the sum of the 10 items within a range of 1–50. Then, the mean (22.32) was calculated as well as used as the cutoff to establish a binary variable, whereby respondents with a score above 22.32 were regarded to have positive acceptability (labelled “0”), while those below and equal to 22.32 were regarded to have negative acceptability (labelled “1”).

#### 3.3.5. Acceptability of Self-Breast Examination (SBE)

Acceptability of the SBE was assessed using a 6-item 5-Likert scale measurement. We calculated the overall score as the sum of the six items within a range of 1–30. Then, the mean (11.81) was calculated as well as used as the cutoff to establish a binary variable that respondents with a score above 11.81 were regarded to have positive acceptability (labelled “0”), while below and equal to 11.81 was regarded to have negative acceptability (labelled “1”).

## 4. Data Processing and Analysis Methods

All the questionnaires were coded, cleaned, and entered into Microsoft Excel. Frequency distribution tables were used to identify and address missing or erroneous data points. Fortunately, after cleaning, the dataset did not contain any missing data. Subsequently, the cleaned data were imported into STATA 18.0, for further analysis.

Descriptive statistics were generated for demographic characteristics, knowledge, attitudes, and BCS elements, using frequency distribution tables. To capture the complete age distribution of the respondents, age was presented using both mean (± SD) and categorical frequencies. To identify the factors associated with BCS uptake among Catholic nuns, a binary logistic regression was performed. This analysis included all variables in the bivariate analysis, adjusted for respondent demographics. All statistical tests were conducted at the 5% significance level.

## 5. Results

### 5.1. Sociodemographic Characteristics

As summarized in Table 1, a total of 385 nuns participated in this study, with an age range of 20–60 years and a mean age of 45.8 (± 15.4) years. The leading age group was 41–50 years old, accounting for 26.2% (*n* = 101) of the study participants. Of the participants, 40% (*n* = 154) had secondary education, 31.7% (*n* = 122) were secondary schoolteachers, and 13.0% (*n* = 50) had pastoral religious qualifications. Of the Catholic nuns, 32.5% (*n* = 125) obtained breast health information from fellow nuns and only 3.4% (*n* = 13) of the participants obtained information from the Internet (Table 1).

### 5.2. Uptake of BCS

**Figure 1:**
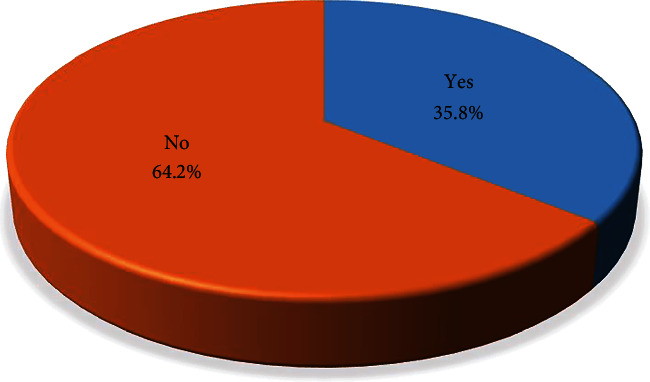
Participant distribution on the uptake of BCS.

The results showed that a total number of 247 (64.2%) (95% CI: 59.2%–68.9%) Catholic nuns had never undergone BCS (see Figure 1).

### 5.3. Knowledge of the Uptake of BCS

This study found a significant knowledge gap regarding BCS among the Catholic nuns. More than half of the 221 (57.4%,95% CI: 52.4%–62.4%) participants had inadequate knowledge about appropriate screening practices. Potential factors contributing to this knowledge gap were identified. A substantial number 336 (87.3%) held misconceptions regarding the recommended starting age for SBE. Additionally, the large majority 306 (79.5%) had never received guidance on performing SBE, and only a small percentage 78 (20.3%) reported relevant educational sessions within the past 2 years. Interestingly, despite these limitations in knowledge and practice, over three-quarters of 290 (75.3%) participants acknowledged the value of SBE for early detection of BC.

### 5.4. Attitudes Towards the Uptake of BCS Among Catholic Nuns

This study identified negative attitudes towards a crucial BCS practice among a significant number of participating Catholic nuns (61.3%, 95% CI: 56.4%–66.2%). More than half 202 (52.5%) strongly disagreed with the statement that prior radiation therapy to the head, neck, or chest increased the risk of BC, and more than half 204 (53.0%) strongly disagreed that monthly SBE could detect breast lumps. More than half 197 (51.2%) of the participants disagreed with annual mammography and CBE.

### 5.5. Acceptability of BCS

Over half of participating Catholic nuns had a negative view on both BCS 133 (55.6%, 95% CI, 50.6%–60.6%) and SBE 133 (52.7%) (95% CI: 47.7%–57.7%). A substantial number of 202 (52.5%) who disagreed with exercise had a reduced risk of developing BC. Half of the 194 (50.4%) participants disagreed that obesity could put them at a higher risk of BC. In addition, one study found that over half of 200 (52.0%) participants disagreed with specific instructions for performing a BSE. Interestingly, 205 (53.3%) agreed on the importance of promoting regular SBE within their congregations.

### 5.6. Factors Associated With the Acceptability and Uptake of BCS Services Among Catholic Nuns

The bivariate analysis indicates that Catholic nuns aged above 60 years were less likely to not perform BCS with a COR of 0.62 (95% CI, 0.39–0.97, *p* = 0.035). Also, catholic nuns who are in the nonhealth field are more likely to not perform BCS with a COR of 1.71 (95%, 1.07–2.74, *p* = 0.025). Likewise, Catholic nuns with negative acceptability of BCS had 1.56 times higher odds (95% CI, 1.02–2.37, *p* = 0.038) of not performing BCS.

During multivariable analysis, it was found that the acceptability of the SBE of catholic nuns significantly influences the acceptability and uptake of BCS. Thus, Catholic nuns who had negative acceptability of SBE were more likely to not perform BCS with an AOR of 1.65 (95% CI, 1.07–2.55, *p* = 0.024) (Table 2).

## 6. Discussion

This study revealed that Catholic nuns had very low acceptance and uptake of BCS practices due to their low knowledge, lack of confidence in how to practice BCS, and lack of breast health services with their congregation. Our findings align with Joseph, Mbuthia, and Kawira [26] who reported a lower BCS rate (27%) among Nigerian Catholic nuns compared to the general population. This disparity may be due to financial constraints and limited access to screening programs within their congregations. The study findings showed that nuns had BCS, which is relatively low considering that they are at high risk. Low BCS practices among nuns have also been reported in other studies [26, 37]. A study conducted by Allen et al. [25] in Massachusetts among Catholic Latinos revealed that BCS uptake was low, at a rate of 24%. Barriers to screening among Latinos have been investigated, including lack of health insurance, concerns about costs, perceived discrimination, inadequate awareness, and lack of provider recommendations [25]. Another study conducted by Wuur et al. [38] in Ghana revealed that the majority of respondents had ever been screened for BC, with only a few having their breasts examined by a healthcare provider (CBE). These findings underscore the need for targeted interventions to address these barriers and improve BCS rates among vulnerable populations, including Catholic nuns.

The current study revealed that only a few Catholic nuns knew how to perform SBE, and the majority did not believe that BC can be hereditary. This finding is similar to that of a study conducted by Okabia et al. [39] in Nigeria, which revealed that only 26.2% of the participants were aware that BC could be inherited in some families. Another study by Yambem and Rahman [40] revealed that of 138 women (46%) who were aware of BSE, 41.3% (*n* = 57) practiced it. A study by Safarpour, Tiyuri, and Mohamadzade [41] noted that only 17.1% of the patients underwent screening examinations. Regarding the best time for checking lumps, most participants admitted that they were unsure about the ideal time for BSE. This aligns with a previous study by Sambanje and Mafuvadze [42] who found that over half of the students lacked knowledge of the optimal time for BSE. Another study conducted in Cameroon by Nde et al. [43] indicated that only 9% of the study participants knew how to perform BSE.

The current findings show that Catholic nuns in the four studied congregations had poor acceptance and uptake of BCS practices. Our findings revealed that most Catholic nuns do not practice BCS. A similar study conducted in Uganda among reverend sisters in Kampala, the largest Archdiocese of the Roman Catholic Church in Uganda, showed that a majority (96.4%) of the respondents did not undergo mammography, 54.1% never practiced BSE, and 34.2% performed it regularly during bedtime [37].

Most of our participants were not aware if the CBE and mammography are useful tools for early diagnosis of BC; moreover, the participants had very low uptake of the CBE procedure, which is a useful tool for BC detection, while most of the participants “disagree,” CBE should not be started within 40 years and above. This study is similar to the study conducted by Odusanya and Olumuyiwa [44] in Nigeria, which revealed that the uptake of screening methods among participants was very low; only 35.0% practiced BSE, only 9.1% had CBE in the past year, and none ever had a mammogram practice [39]. The two most prevalent reasons women reported not receiving CBEs were a lack of awareness about their necessity and not experiencing any breast issues. These shared findings underscore the urgent need for improved education and outreach programs to raise awareness of the significance of early detection methods, such as CBEs and mammograms, in the fight against BC.

In another study conducted by Dang, Lee, and Tran [45] in Southeast Asia, only 36.1% had undergone mammography. Another study conducted in the KSA reported that 92% of women aged ≥ 50 years had never undergone mammogram screening [46]. These low levels of screening and infrequent practices of regular BCS likely reflect a general lack of awareness and poor knowledge of BCS within the population. Another similar study conducted in the Oyo state of Nigerian nuns reported poor uptake of CBE, and only 28.8% had a CBE [47]. Another study by Opoku, Benwell, and Yarney [48] in Ghana revealed that respondents' attitudes included fear of the disease, which was linked to death in most cases, denial and guilt, and supernatural attributes. The self-reported BCS rate (BSE, 32%; CBE, 12%; and mammography, 2%) was poor; however, higher educational levels were significantly associated with BCS practices.

The findings of our study suggest that negative acceptability and low uptake have negative effects on BCS. Participants with negative acceptance and uptake were more likely to not undergo BCS. Our finding is similar to another report that a lack of awareness regarding the early detection of BC among African women living in SSA was one reason for the low uptake of BCS practice [49]. Barriers to BCS have been shown to exist in many countries. The greatest barriers identified against BCS uptake include lack of accessibility, lack of knowledge of BCS, and unavailability of updated breast health interventions within congregations [37].

## 7. Conclusion

This study reveals a low uptake of BCS among Catholic nuns. Furthermore, our findings highlight significant gaps in their knowledge, acceptance, and practices related to breast health. These findings underscore the urgent need for multifaceted interventions tailored to this specific population to address the barriers hindering BCS and improve health outcomes.

## 8. Recommendation

We recommend both health education and promotional intervention among this special group of catholic nuns. The interventions could include detailed and regular sensitization and awareness programs specifically designed for Catholic nuns. Crucially, these programs should emphasize the availability of screening programs, along with the importance and necessity of participation. The primary aim should be to address the identified barriers and dispel any misconceptions related to BCS. Therefore, it is essential to create adequate awareness through targeted educational intervention. Educating Catholic reverend sisters through these programs can empower them to make informed decisions about their breast health.

## Figures and Tables

**Table 1:** Social demographical characteristics.

**Variable**	**N** ** (%)**
Age group	
20–30	89 (23.1)
31–40	55 (14.3)
41–50	101 (26.2)
51–60	69 (17.9)
61+ and above	71 (18.4)
Education level	
Primary education	79 (20.5)
Secondary education	154 (40.0)
Degree	139 (36.1)
Master and above	13 (3.4)
Occupation	
Primary teacher	11 (2.9)
Secondary teacher	122 (31.7)
Pastoral religious	50 (12.9)
Cooker	34 (8.8)
Spiritual counselor	28 (7.3)
Nurse	40 (10.4)
Doctor	19 (4.9)
Clinical officer	38 (9.9)
Other	43 (11.2)
Working experience	
1–5 years	100 (25.9)
5–10 years	67 (17.4)
11–15 years	81 (21.0)
15 years and above	137 (35.6)
Source of information about breast cancer	
Family member	44 (11.4)
Sisters	125 (32.5)
Health providers	92 (23.9)
Tv media	111 (28.8)
Other sources like the Internet	13 (3.4)

**Table 2:** Factors associated with uptake of breast cancer screening among Catholic nuns.

**Variable**	**Ever performed BCS**		**COR (95% CI)**	**p** ** value**	**AOR (95% CI)**	**p** ** value**
	**Yes** **N** ** (%)**	**No** **N** ** (%)**				
Age category						
Below 60 years	42 (29.2)	102 (70.8)	1		1	
Above 60 years	96 (39.8)	145 (60.2)	0.62, 0.39–0.97	0.035	0.73, 0.46–1.17	0.197
Education background						
Primary education	28 (35.4)	51 (64.6)	1		1	
Secondary education	49 (31.8)	105 (68.2)	1.18, 0.66–2.09	0.578	1.28, 0.71–2.31	0.418
Collage and above	61 (40.1)	91 (59.9)	0.82, 0.47–1.44	0.488	0.91, 0.50–1.64	0.755
Occupation status						
Health field	44 (45.4)	53 (54.6)	1		1	
Nonhealth field	94 (32.6)	194 (67.4)	1.71, 1.07–2.74	0.025	1.50, 0.92–2.46	0.105
Knowledge of breast cancer screening						
Adequate knowledge	63 (38.4)	101 (61.6)	1		1	
Inadequate knowledge	75 (33.9)	146 (66.1)	1.21, 0.79–1.85	0.365	1.21, 0.78–1.88	0.384
Attitude towards breast cancer screening						
Positive attitude	62 (41.6)	87 (58.4)	1		1	
Negative attitude	76 (32.2)	160 (67.8)	1.50, 0.98–2.29	0.061	1.42, 0.91–2.20	0.118
Acceptability of breast cancer screening						
Positive acceptability	57 (33.3)	114 (66.7)	1		1	
Negative acceptability	81 (37.9)	133 (62.1)	0.82, 0.54–1.25	0.359	0.79, 0.51–1.23	0.297
Acceptability of self-breast examination						
Positive acceptability	75 (41.2)	107 (58.8)	1		1	
Negative acceptability	63 (31.0)	140 (69.0)	1.56, 1.02–2.37	0.038	1.65, 1.07–2.55	0.024

Abbreviations: AOR = adjusted odds ratio; BCS = breast cancer screening; CI = confidence interval; COR = crude odds ratio; *p* value = probability value.

## Data Availability

The datasets generated and/or analyzed during the current study are available from the corresponding author upon reasonable request.

## Nomenclature

AOR: adjusted odds ratio
BC: breast cancer
BCS: breast cancer screening
BSE: breast self-examination
CBE: clinical breast examination
CI: confidence interval
COR: crude odds ratio
*p* value: probability value
